# Synthetic nanoparticles for delivery of radioisotopes and radiosensitizers in cancer therapy

**DOI:** 10.1186/s12645-016-0022-9

**Published:** 2016-11-16

**Authors:** Jun Zhao, Min Zhou, Chun Li

**Affiliations:** 1 Department of Cancer Systems Imaging, The University of Texas MD Anderson Cancer Center, 1881 East Road, Houston, TX 77054 USA; 2 Institute of Translational Medicine, Zhejiang University, Hangzhou, 310009 Zhejiang China

**Keywords:** Nanoparticles, Radiotherapy, Radiosensitization, Radioisotopes

## Abstract

Radiotherapy has been, and will continue to be, a critical modality to treat cancer. Since the discovery of radiation-induced cytotoxicity in the late 19th century, both external and internal radiation sources have provided tremendous benefits to extend the life of cancer patients. Despite the dramatic improvement of radiation techniques, however, one challenge persists to limit the anti-tumor efficacy of radiotherapy, which is to maximize the deposited dose in tumor while sparing the rest of the healthy vital organs. Nanomedicine has stepped into the spotlight of cancer diagnosis and therapy during the past decades. Nanoparticles can potentiate radiotherapy by specifically delivering radionuclides or radiosensitizers into tumors, therefore enhancing the efficacy while alleviating the toxicity of radiotherapy. This paper reviews recent advances in synthetic nanoparticles for radiotherapy and radiosensitization, with a focus on the enhancement of in vivo anti-tumor activities. We also provide a brief discussion on radiation-associated toxicities as this is an area that, up to date, has been largely missing in the literature and should be closely examined in future studies involving nanoparticle-mediated radiosensitization.

## Background

The cytotoxic effects of ionizing radiation were first observed in 1895, when Wilhelm Röntgen intentionally subjected his finger to X-rays. Burns developed on the irradiated finger shortly after the exposure (Assmus 1995). Since then, ionizing radiation using both external and internal radiation sources has become one of the three pillars of anti-cancer treatments along with surgery and chemotherapy (Zoller et al. 2009). Nevertheless, contemporary radiation techniques are frequently challenged by the need to deposit as much energy as possible in tumor regions while minimizing the collateral damage to normal tissues. Indeed, toxic side-effects are often the dose-limiting factors in many cases, and can prevent the further escalation of radiation dose (DeNardo and Denardo 2006).

During the past several decades, nanomedicine has evolved into a promising player in cancer diagnosis and therapy (Retif et al. 2015; Li 2014). Nanoparticles can enhance the efficacy of radiotherapy through several mechanisms. The “targeted” nanoparticles, via either passive or active mechanisms, can selectively deliver radioisotopes into tumors (Eetezadi et al. 2015). Radiosensitizers can also be delivered to solid tumors through the use of nano-carriers to make tumors more vulnerable to external radiation. In addition, nanoparticles are ideal platforms to incorporate multiple functions and enable multimodality therapy. For example, multiple chemotherapy drugs can be loaded in the same nanoparticles to achieve a synergistic anti-tumor effect with radiotherapy. Imaging capabilities may also be integrated into nanoparticle design to provide image guidance (Phillips et al. 2014).

### External radiation sources

External beam radiation treatment (EBRT) utilizes an external linear accelerator to generate high-energy X-rays, and delivers them to tumors. The photon energy of X-rays ranges from kilo- to mega-volts. Compared to the kilovoltage X-rays, the megavoltage X-rays can irradiate deep-seated tumors with minimal burning of superficial tissues, and therefore are widely used in patients (Wang et al. 2010). Along with the development of computer and electronic techniques within the past three decades, the way of delivering radiation beams has evolved significantly. The contemporary techniques include 3D-conformal radiotherapy, intensity-modulated radiotherapy (IMRT), and image-guided radiotherapy (DeNardo and Denardo 2006). Compared to conventional EBRT, IMRT delivers radiation dose with increased conformality, therefore limiting the dose exposure to normal organs (Samuelian et al. 2012). In a retrospective study by Samuelian et al. (Samuelian et al. 2012), 62% of patients experienced ≥grade-2 acute gastrointestinal toxicity after conventional EBRT, while only 32% of patients had the same side-effects following IMRT (*p* = 0.006). Stereotactic body radiotherapy (SBRT) is another novel technique that can deliver a large radiation dose to tumor while keeping a plummet of dose at the peripheral of target regions. In this way, the volume of irradiated normal tissues is minimized. SBRT has been successfully used in treating lung, liver, spine, kidney, and pancreatic cancers (Pollom et al. 2015).

### Internal radiation sources

Free metal ions are rarely injected by themselves due to their unfavorable biodistribution profiles. For example, free ^90^Y ions tend to deposit in bone (~50% of injected radioactivity) and cause bone toxicities (Ando et al. 1989). As a result, radioisotopes are often conjugated to carriers to achieve a tumor-specific accumulation. For instance, ^90^Y and ^177^Lu are frequently conjugated to tumor-specific peptides in the peptide receptor radionuclide therapy (PRRT) (van Essen et al. 2009). The anti-tumor efficacy of radioisotopes is affected by their tissue penetration: long-penetrating radioisotopes (e.g., ^90^Y) are more suitable for larger tumors, whereas short-ranged ones (e.g., ^177^Lu) can better treat micro-metastases (Villard et al. 2012). In addition to β emitters, α emitters and Auger emitters also have been evaluated in cancer therapy due to their promising energy transfer properties. For example, ^211^Astatine is a synthetic α emitter with a mean linear energy transfer value optimal for inducing DNA double-strand breaks (Langen et al. 2015). Auger electrons (Kassis 2004), on the other hand, are low-energy electrons generated by the radioisotopes that decay by electron capture and/or internal conversion (e.g., ^125^Iodine, ^123^Iodine, and ^77^Bromine). Such transitions of the inner-shell electrons result in a characteristic atomic X-ray photon, or low-energy and short-range mono-energetic electrons (collectively known as Auger electrons). Auger electrons have uniquely high values of linear energy transfer (~26 keV/µm) within several cubic-nanometers from the site of decay. They can generate far more damage to DNA strands than those high-energy electrons. Several radioisotopes commonly used in radiotherapy are listed in Table 1.

**Table 1:** Properties of commonly used radioisotopes in radiotherapy

Radionuclide	Half-life (Hour)	Emission type	Energy	Range in tissue	Ref.
^111^Indium (^111^In)	67.9	Auger	2.5–25 keV	10 µm	(Giovacchini et al. 2012)
		γ	173–247 keV		
^177^Lutetium (^177^Lu)	161.5	β^−^	*E*_max_ = 0.497 MeV *E*_mean_ = 0.149 MeV	2 mm	(Giovacchini et al. 2012; Nilsson et al. 2011)
		γ	113 ~ 321 keV		
^188^Rhenium (^188^Re)	17	β^−^	2.12 MeV	3.5 mm	(Lin et al. 2014; Phaeton et al. 2016)
		γ	155 keV		
^90^Yttrium (^90^Y)	64.1	β^−^	*E*_max_ = 2.28 MeV *E*_mean_ = 0.935 MeV	4.1–11.3 mm	(Giovacchini et al. 2012; Kennedy 2014)

## Radioisotope-loaded nanoparticles for internal radiation

### Radiolabeling of nanoparticles via chelators

Bifunctional chelators are normally needed to introduce radioisotopes to nanoparticles to achieve high radiolabeling stability. Since many excellent reviews have been published in this area (DeNardo and Denardo 2006; Liu 2008; Anderson and Welch 1999; Pohlman et al. 2006), only a brief discussion is presented in this section.

**Fig. 1:**
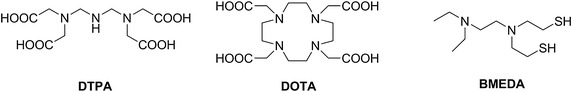
Examples of radioisotope chelators (Kennedy 2014): diethylenetriaminepentaacetic acid (DTPA), 1,4,7,10-tetraazacyclododecane-1,4,7,10-tetraacetic acid (DOTA), and N,N-bis(2-mercaptoethyl) –N’,N’-diethylethylenediamine (BMEDA)

The chemical structures of several chelators are listed in Fig. 1. Diethylenetriaminepentaacetic acid (DTPA) exhibits high efficiency of radiolabeling under mild conditions, making it an attractive candidate to label nanoparticles sensitive to heat or pH. However, DTPA chelation is kinetically labile, and may cause the dissociation of radioisotopes in vivo (Camera et al. 1994). As an example, Werner et al. (2011) developed biodegradable PLGA–lecithin–PEG core–shell nanoparticles that encapsulated paclitaxel in the core and chelated ^90^Y on the surface via DTPA. Folic acid was conjugated to the surface of these nanoparticles as the targeting ligand. The resultant nanoparticles were 75 ± 10 nm in size and −35 ± 5 mV in surface charge. In an orthotopic ovarian cancer xenograft model, mice receiving the nanoparticles with both paclitaxel and ^90^Y at a dose of 500 µg nanoparticles/mouse (20 µg paclitaxel and 1.85 MBq ^90^Y per mouse) showed significant survival advantage over those receiving monotherapies.

1,4,7,10-Tetraazacyclododecane-1,4,7,10-tetraacetic acid (DOTA) is a macrocyclic chelator with kinetic inertness after chelation with radionuclides. However, the labeling yield can be affected by many parameters such as the DOTA concentration, pH, reaction temperature, and heating time. Heating (e.g., >50 °C) is usually required for a high labeling efficiency. Under such conditions, the denaturing of bioactive antibodies or disruption of nanoparticles may be a concern (Li et al. 1994). Wilson et al. (2012) conjugated ^177^Lu^3+^ to the surface of tri-gadolinium nitride C_80_ endohedral metallofullerene nanoparticles (Gd_3_N@C_80_) via DOTA chelator. Previously, Gd_3_N@C_80_ nanoparticles have been examined as highly efficient contrast agents for magnetic resonance imaging (Fatouros et al. 2006). The radiolabeling yield for ^177^Lu^3+^ was 50% despite 12 h of reaction at 40 °C. The anti-tumor activity of ^177^Lu^3+^-labeled Gd_3_N@C_80_ nanoparticles were evaluated in two orthotopic glioblastoma models (U87MG and GBM12) in mice after local–regional injection via convection-enhanced delivery (CED). During CED, a catheter is directly placed into tumor via stereotactic methods, followed by a slow injection of anti-cancer agents under a positive pressure gradient. Compared to the simple intratumoral injection, nanoparticles injected under the CED setting had a more uniform distribution inside the tumor. Doses of 0.25–1.35 MBq significantly increased animal survival in both glioma xenograft models. The positive response was attributed to the short penetration distance (0.7 mm) of ^177^Lu, which deposited the majority of radiation energy on tumor cells.

N,N-Bis(2-mercaptoethyl) –N’,N’-diethylethylenediamine (BMEDA) is frequently used to chelate ^188^Re (Bao et al. 2003; Chang et al. 2007), which shows potentials in cancer diagnosis and therapy because it emits both γ rays (155 keV) and β particles (2.12 MeV) (Deutsch et al. 1986). Huang et al. (2011) prepared ^188^Re-BMEDA-loaded liposomes with a radiolabeling efficiency of 88.0 ± 2.8%. The resulting liposomes were 80.3 ± 1.1 nm in average diameter and −1.44 mV in ζ potential. Seventy-four percent of ^188^Re was retained in liposomes after 72 h of incubation in serum. In an orthotopic rat glioma model, it was found that about 1.95% of injected dose was retained in each gram of tumor tissue (1.95% ID/g) at 24 h after intravenous injection. In comparison, the uptake in normal brain was less than 0.08% ID/g. Lin et al. (2014) prepared a similar liposomal formulation to treat non-small cell lung cancer (NSCLC) in an orthotopic tumor model. Compared to the small-molecule complex ^188^Re-BMEDA, ^188^Re-liposomes exhibited higher blood retention and higher tumor uptake. A single injection of ^188^Re-liposomes at a dose of 23.7 MBq/mouse effectively delayed tumor growth.

Other labeling techniques have also been used for introducing ^188^Re to nanoparticles. Vanpouille-Box et al. (2011) prepared ^188^Re-labeled lipid nanocapsules using dithiobenzoate. In this technique, perrhenate ^188^ReO_4_^−^ ion was first reduced by a cocktail of SnCl_2_/potassium oxalate/ascorbic acid/sodium gluconate, and then chelated by sodium dithiobenzoate to form a hydrophobic complex of ^188^Re(III)(PhCS_3_)_2_(PhCS_2_), abbreviated as ^188^Re-SSS (Lepareur et al. 2004). ^188^Re-SSS was then physically loaded into lipid nanocapsules, which were locally injected into 9L rat glioma in immunocompetent Fisher 344 rats by using CED technique at 2.8 MBq/injection for two injections. The nanocapsules significantly prolonged the tumor retention of ^188^Re-SSS: more than 75% ID/g remained in brain at 96 h post injection. In contrast, 70% of injected ^188^ReO_4_^−^ had been cleared from the body during the same time period. A promising cure rate of 83% was observed using an optimized treatment schedule. It is interested to note that ^188^Re-SSS-loaded lipid nanocapsules also suppress the growth of secondary tumors, suggesting a possible role of therapy-induced anti-tumor immune response.

The naturally existent porphyrins have exceptionally high affinity with metal ions and display intrinsic fluorescence (Smith and Gouterman 1968; Bases et al. 1958). Liu et al. (2013) labeled ^64^Cu to a porphyrin-based liposome (^64^Cu-porphysomes) to delineate prostate tumors in orthotopic models by using positron emission tomography (PET). At 24 h after injection, the tumor uptake was 6.83 ± 1.08% ID/g in PC-3 and 4.81 ± 2.06% ID/g in 22RV1 prostate tumors in mice. Notably, micro-metastases as small as 1.4 mm were detected in bone. Since tumors at this size have limited angiogenic blood vessels, tumor uptake in small metastatic lesions may be facilitated by mechanisms other than the classical enhanced permeability and retention effect. Substitution of ^64^Cu with therapeutic radioisotopes may render this class of nanoparticles suitable nano-carriers for treatment of micro-metastatic lesions.

### Chelator-free radiolabeling of nanoparticles

Although it has been extensively used at both preclinical and clinical stages, chelator-based radiolabeling still faces some limitations. There is no single chelator that binds to all isotopes with thermodynamic and kinetic stability. In many cases, the optimal chelator for an isotope needs to be empirically determined. Moreover, the in vivo stability of chelation can be compromised by endogenous protein trans-chelation, leading to the dissociation of isotopes from nanoparticles (Boswell et al. 2004).

**Fig. 2:**
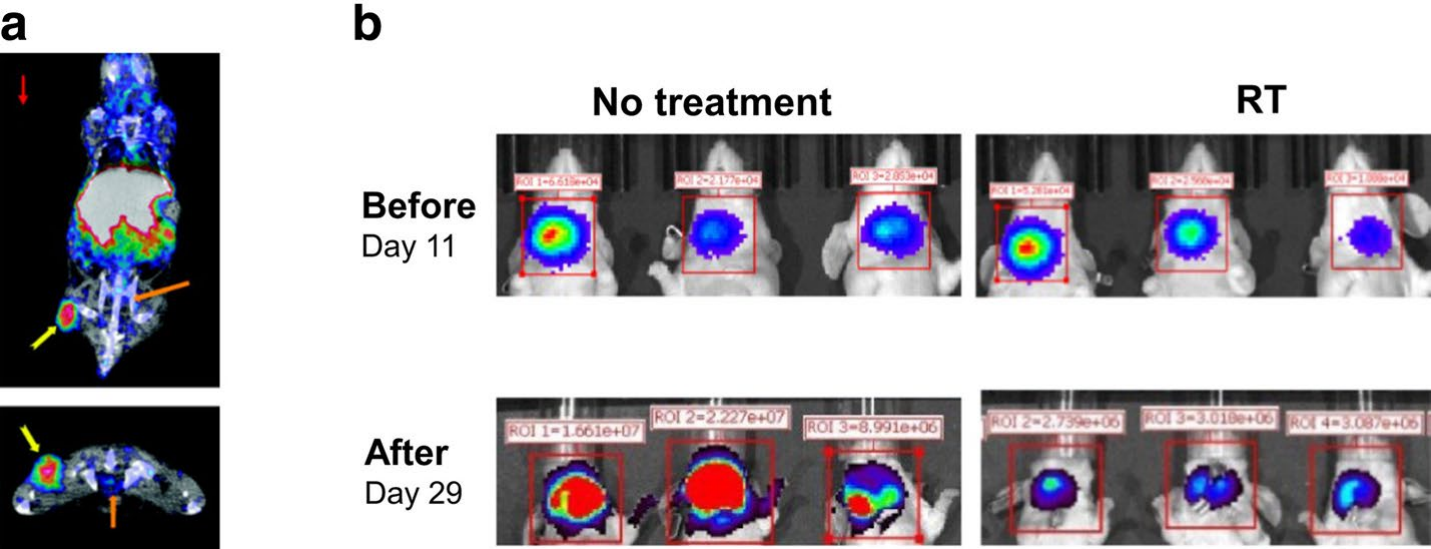
[^64^Cu]–CuS nanoparticles were used for both PET imaging (**a**) and radiotherapy (**b**). [reused with permission from references (Zhou et al. 2010, 2015a)]

We are among the first to introduce the concept of chelator-free radioactive nanoparticles taking advantage of radioisotopes and their non-radioactive isotopes of the same element as integral components of nanoparticles. This is exemplified by the synthesis of chelator-free ^64^Cu-doped copper sulfide nanoparticles ([^64^Cu]-CuS NPs) (Zhou et al. 2010). ^64^Cu is a unique radioisotope suitable for both PET imaging and radiotherapy, because it emits 0.653 MeV positron (17.8%) and 0.579 MeV beta particles (38.4%) at a half-life of 12.7 h. We have synthesized highly stable [^64^Cu]-CuS NPs with high radiochemical yield (Zhou et al. 2010). These nanoparticles were 11.7 nm in size with citrate coating, and 31.6 nm with polyethylene glycol (PEG) coating, and displayed strong absorption of near-infrared light. PET/CT imaging clearly delineated the U87 glioma xenograft at 24 h post injection, with a tumor uptake value of 7.6 ± 1.4% ID/g (Fig. 2). In subsequent studies, we demonstrated that PEG-[^64^Cu]-CuS could be used to treat anaplastic thyroid cancer (Zhou et al. 2015a). Intratumoral injection of PEG-[^64^Cu]-CuS at 7.4 MBq/mouse significantly delayed tumor growth compared to the non-treatment control (*p* < 0.0053), while no significant systemic toxicity was observed.

Black et al. (2014) added H^198^AuCl_4_ to the starting materials and directly incorporated ^198^Au into the crystal lattice of Au nanostructures. The specific activity was tuned by changing the ration of H^198^AuCl_4_ to HAuCl_4_. The γ emission from ^198^Au enabled single-photon emission computed tomography (SPECT), while the β emission produced luminescence imaging via Cerenkov radiation.

Later work extended the concept of chelator-free radiolabeling to nanoparticles with radioisotopes that are not part of the building components of the nanoparticle. For example, Shaffer et al. (2015) reported silica nanoparticles for a generalized immobilization of radionuclides, including ^89^Zr, ^68^Ga, ^111^In, ^90^Y, ^177^Lu, and ^64^Cu. The radionuclides were bound by the oxygen atoms of the silica lattice. Radiolabeling was performed in a range of pH values (pH = 5.7–8.8) at 70 °C for 15 min, to give an efficiency >99% and a specificity of 3.7 TBq/µmol. Radovic et al. (2015) absorbed ^90^Y^3+^ ions onto the negatively charged surface of Fe_3_O_4_ nanoparticles, with a labeling yield >99%. However, 5–20% of the ^90^Y dissociated after 1 week. Liu et al. (2015) absorbed ^64^Cu on the surface of MoS_2_ nanosheets through the high affinity between Cu and S ions; 70–85% labeling efficiency was achieved by shaking at 37 °C for 1 h. The labeling was stable (>85% retention) during serum incubation at 37 °C for up to 48 h. Chelator-free labeling of nanoparticles by surface absorption is in general less stable compared to labeling with radioisotopes to be integrated to the matrix of the nanoparticles.

Sun et al. (2014) prepared ^64^Cu-doped CdSe/ZnS core/shell quantum dots via cation exchange reaction in organic phase. After 1 h of incubation at 60 °C, the labeling efficiency was almost 100% with a specific activity of 7.4 MBq/mg of quantum dots. The shape and size of quantum dots were preserved after radiolabeling. The labeling was highly stable during serum incubation at 37 °C for up to 48 h. Notably, the encapsulated ^64^Cu enabled the Cerenkov luminescence and irradiated the quantum dots to generate luminescence. A similar method was used by Guo et al. (2015) for the preparation of ^64^Cu-labeled CuInS/ZnS quantum dots. In another study, Sun et al. (2014) prepared ^64^Cu-coated Au nanoparticles by reducing ^64^Cu^2+^ with hydrazine in the presence of Au nanoparticles. The formed ^64^Cu element deposited on Au surface to achieve a radiolabeling up to 100% efficiency. Various Au nanoparticles were successfully labeled without changes in morphology or size.

Chloramine T oxidation is a common method for radioiodination of antibodies (Yamada et al. 2000). During the reaction, iodide is first oxidized to iodine monochloride, which then reacts with aromatic rings to form an iodine-tagged aromatic ring. Chloramine T oxidation has been used to label PEG-coated graphene nanosheets (PEG-GNS). Yang et al. (2011) prepared ^125^I-PEG-GNS with a labeling efficiency between 50 and 60% and a specific activity of 7.4 MBq/mg of NGS. Less than 5% of ^125^I detached after 15 days of incubation at 37 °C in saline or serum. Chen et al. (2015) used ^131^I-labeled PEG-GNS for the radiation/photothermal therapy in a 4T1 murine breast cancer model. The resultant ^131^I-PEG-GNS was 50 nm in size and 3 nm in height. A majority of nanoparticles were entrapped in liver and spleen (15–20 ID %g ) with a tumor uptake value of 5% ID/g at 48 h after intravenous injection at a dose of 100 mg/kg (7.4 MBq/mouse). Radiotherapy reduced tumor volume by about 70% at 18 days after treatment, while the combination therapy eliminated tumor in 4 out of 5 mice. No significant toxicities were observed in terms of liver, spleen, hematological toxicities, or body weight loss.

### Neutron-activated nanoparticles

^166^Ho is an attractive radionuclide for radiotherapy by emitting both gamma photons (81 keV, 6.6%) and high-energy β particles (maximum energy at 1.84 MeV) at a half-life of 26.8 h. It also provides contrast to CT and MRI due to its high attenuation coefficient and paramagnetic properties, respectively (Seevinck et al. 2007). ^166^Ho-labeled nanoparticles can be prepared through the neutron activation of the stable isotope ^165^Ho. The yield of ^166^Ho conversion is proportional to the duration of neutron activation. Di Pasqua et al. (Di Pasqua et al. 2013) doped MCM-41-type mesoporous silica nanoparticles (MSN) with ^165^Ho(AcAc)_3_, which was then converted to ^166^Ho by neutron activation. The resultant ^166^Ho-MSN nanoparticles were 80–100 nm in size and −49.2 ± 6.0 mV in surface charge, with a specific activity of 12 MBq/mg. ^166^Ho-MSN was evaluated in an orthotopic SKOV3 ovarian cancer xenograft model by intraperitoneal injection. The tumor uptake (ID%/g) was 32.8 ± 8.1% at 24 h post injection, and reached 81 ± 7.5% 1 week after. It should be noted that the tumor uptake value reported here was remarkably high compared to many other radioisotope-labeled nanoparticles. However, no explanation was given for the underlying tumor-homing mechanism, which may be in part attributed to the intraperitoneal injection route. In terms of anti-tumor efficacy, one injection of 4 MBq ^166^Ho-MSN reduced the tumor metabolism activity by 50% compared to control. Ninety-seven percent of the treated mice were alive at 75 days post-treatment, which was significantly higher than the groups without treatment or receiving free ^166^Ho alone. In a follow-up study, Munaweera et al. (2015) prepared ^165^Ho-doped garnet magnetic nanoparticles and loaded them with platinum-based radiosensitizers. The formed nanoparticles were 40.7 ± 16.4 nm in length and 26.9 ± 8.0 nm in width, with typical ferromagnetic behaviors. Up to 6.2% (by weight) of platinum was loaded, and the specific activity was 9.25 MBq/mg.

## Biological considerations for radiotherapy

### Administration routes of nanoparticles

In preclinical studies, nanoparticles are commonly administered via intravenous or intratumoral injection. Compared to intravenous injection, intratumoral injection of nanoparticles can directly deposit large doses to tumor site, without the toxicity to other organs. The theoretical simulations by Sinha et al. (2015) suggested that intratumoral injection of AuNPs provided better radiosensitization effects than intravenous injection. However, intratumoral injection is considered as a local therapy and is not suitable for treating disseminated disease. Due to the presence of brain–blood barrier in brain tumors, convection-enhanced delivery (CED) can provide a more uniform intratumoral distribution of nanoparticles (Fatouros et al. 2006). For pulmonary malignancies, on the other hand, inhalation is a viable option (Muralidharan et al. 2015). For ovarian cancer, intraperitoneal injection is sometimes used to increase exposure of tumors to the radioactive nanoparticles in the abdomen cavity (Di Pasqua et al. 2013).

In addition to tissue distribution, the radiosensitization effects of nanoparticles depend on their concentration as well as distribution inside cells. McQuaid et al. (2016) studied the correlation between intracellular distribution of gold nanoparticles and the range of DNA-damaging electrons produced during radiation. Chithrani et al. (2010) showed that AuNPs of 50 nm in size were optimal for cellular uptake and therefore more potent than both larger and smaller AuNPs in terms of radiosensitization. It is known that AuNPs, under radiation, can generate secondary electrons and damage DNAs within 30 nm range (Zheng et al. 2008). Therefore, intranuclear AuNPs can better induce DNA damage than the cytoplasmic ones. Oh et al. (2011) examined the intracellular distribution of gold nanoparticles (AuNPs). Small AuNPs (~2.4 nm) localized in nucleus, while larger ones (5.5–8.2 nm) retained in the cytoplasm in a perinuclear manner. Zhang et al. (2012) compared the radiosensitization effect of PEG-coated AuNPs with sizes of 4.8, 12.1, 27.3 and 46.6 nm. Transmission electron microscopy (TEM) studies found that 4.8- and 46.6-nm AuNPs formed large aggregates when taken by HeLa cells, while the 12.1- and 27.3-nm AuNPs had more uniform distribution. The in vivo biodistribution study revealed that the 12.1-nm AuNPs had the highest tumor uptake. Since the radiosensitization effect is favored by a high intratumoral concentration of AuNPs, the best radiosensitization effect was achieved by the 12.7-nm AuNPs. Rima et al. (2013) examined the intracellular delivery of sub-5 nm gadolinium-based nanoparticles in head and neck squamous cell carcinoma cells. TEM images established that the nanoparticles were internalized via passive diffusion and micropinocytosis, while the latter led to a successful radiosensitization in cell culture. In a follow-up study, Stefancikova et al. (2014) revealed that the gadolinium-based nanoparticles co-localized with lysosomes in U87 cells and still provide radio-enhancement under gamma irradiation.

### In vivo dosimetry of nanoparticle-bound radionuclides

The radiation energy absorbed by tumor or normal tissues is an important predictor to the biological responses in each tissue (Zoller et al. 2009). The absorbed dose, a quantification of such energy, is defined as the energy absorbed per unit mass of tissue (Sgouros 2005). In addition, the tissue responses are also affected by the rate of dose delivery, the type of radiation sources (e.g., α, β, or Auger particles), the radiobiological characteristic of each tissue, as well as the treatment history of patients (Sgouros 2005). Since most nanoparticle-bound radionuclides are used inside the body, a brief introduction to the dosimetry of internal emitters will be discussed in this section.

Absorbed dose (*D*) is the appropriate term of dosimetry in practice, defined as the energy (*E*) absorbed by the tissue, divided by the tissue mass (*M*):

1 $$D = \frac{E}{M} = \frac{{\overset{\lower0.5em\hbox{$\smash{\scriptscriptstyle\smile}$}}{A} \times \Delta \times \varphi }}{M} = \overset{\lower0.5em\hbox{$\smash{\scriptscriptstyle\smile}$}}{A} \times S; \quad where \;\; S = \frac{\Delta \times \varphi }{M},$$

where *E* = number of radionuclide disintegrations in a defined volume (*Ă*) × energy emitted per disintegration (Δ) × fraction of energy absorbed by the tissue mass ($\varphi$). The first term, *Ă*, depends on the half-lives of radionuclides as well as their spatial and temporal distribution. In clinics, the radioactivity from the region of interest is recorded by imaging or sampling, and plotted into a curve against time. The integral of this activity–time curve give the value for *Ă*. The second term, Δ, is related to the emission type, and can be derived from standard dosimetry tables (Browne et al. 1986). The last term, $\varphi$, accounts for the fraction of energy absorbed in the target region that is emitted from the source tissue, and commonly denoted as $\varphi$_target←source_. $\varphi$ is generally derived from Monte Carlo calculations (Snyder et al. 1969). In practice, Δ, $\varphi$ and *M* are combined into one parameter *S*, and the total absorbed dose (*D*_T_) is the sum of doses contributed by different sources:

$$D_{\text{T}} = \overset{\lower0.5em\hbox{$\smash{\scriptscriptstyle\smile}$}}{A}_{S1} \times S_{{{\text{T}} \leftarrow S1}} + \overset{\lower0.5em\hbox{$\smash{\scriptscriptstyle\smile}$}}{A}_{S2} \times S_{{{\text{T}} \leftarrow S2}} + \overset{\lower0.5em\hbox{$\smash{\scriptscriptstyle\smile}$}}{A}_{S3} \times S_{{{\text{T}} \leftarrow S3}} \ldots$$

The list of *S* values can be found from pamphlets published by the Committee on Medical Internal Radiation. However, it should be noted that due to the irregular geometry of tumor, the *S* values generated from the idealized tables may provide erratic information of absorbed doses. Advanced imaging techniques and mathematical simulations have been developed for more accurate estimation (Sgouros 2005).

### Radiation-induced toxicity in healthy organs

Few studies report nanoparticle-mediated radiotoxicity to normal organs. Since nanoparticles are largely distributed to organs of the mononuclear phagocytic system or kidney, we will briefly discuss the general radiotoxicity of liver and kidney.

### Liver

In general, the whole liver can be safely irradiated with up to 30–35 Gy (Lawrence et al. 1995). Higher doses may cause subacute toxicity at 4–8 weeks post radiation, commonly known as the veno-occlusive disease (Fajardo and Colby 1980). It is characterized by congestions in the central portion of liver lobes due to the entrapped erythrocytes, as well as the obstructed sub-lobular veins by collagen fibers. Collagen proliferation may also obstruct some small portal veins toward the end period of subacute damage. Although liver could heal over time after radiation, the asymptomatic chronic injuries may still persist up to 6 years post radiation, such as the distorted structures and fibrosis of liver lobules and veins. Such chronic lesions may be associated with chronic radiation hepatitis in clinics (Lewin and Millis 1973).

### Kidney

Radiation-induced kidney damage can be characterized into acute and chronic nephropathy. The acute symptoms, such as proteinuria, hypertension, heart failure, azotemia, and anemia, reflect the pathological changes in kidney including atrophy, tubulointerstitial scarring, mesangiolysis, and thrombotic microangiopathy. The chronic symptoms, on the other hand, are characterized by the loss of mass and functions in kidney (Moll et al. 2001; Behr et al. 1999). In a follow-up survey, Valkema et al. (2005) observed a sustained decline of creatinine clearance in patients for up to 5.4 years after receiving PRRT.

Kidney can be safely treated with 15–17 Gy of EBRT in 2-Gy fractions. In case of nanoparticle-bound radionuclides, however, the threshold dose may be significantly different; because, both clearance and re-absorption of radionuclides can occur in kidney. Although currently few studies discuss the renal toxicity caused by radiolabeled nanoparticles, the PRRT-induced kidney damages have been reported (Giovacchini et al. 2012; Vegt et al. 2010). For instance, Svensson et al. (2012) showed that proximal tubulars of nude mice can be damaged by ^177^Lu-DOTA-Tyr^3^-octreotate above 24 Gy.

**Fig. 3:**
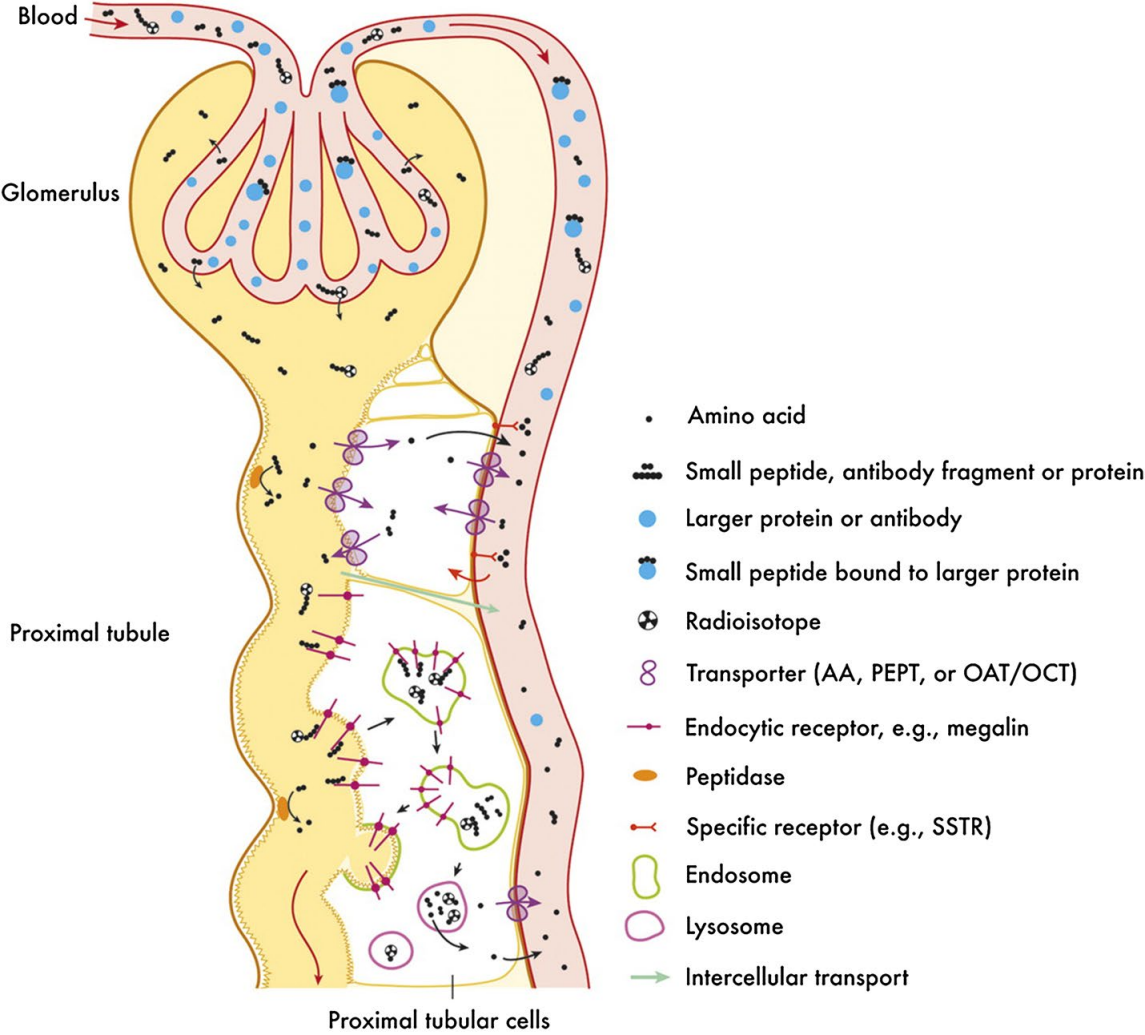
Clearance and re-absorption of peptides and small molecules in kidney. Used with permission from Ref. (Behr et al. 1999). This research was originally published in [71], by the Society of Nuclear Medicine and Molecular Imaging, Inc

Figure 3 shows the trafficking of peptides and small molecules in kidney. It is known that molecules less than 1.8 nm are rapidly filtered through the glomerular membranes and enter the proximal tubules, while those larger than 4–6 nm are mostly retained in blood circulation (Vegt et al. 2010). The filtrate inside proximal tubules, however, can be re-absorbed into blood circulation via active or passive transport. Oligopeptides can be hydrolyzed at the brush border of proximal tubular cells, and re-absorbed by transporters. Large peptides or proteins, on the other hand, are mainly absorbed by receptor-mediated endocytosis (Christensen and Verroust 2002; de Jong et al. 2005). It should be noted that some radiolabeled metabolites cannot escape the lysosomes of tubular cells, and thus reside in kidney and potentially cause radiation toxicity.

Renal toxicities can be alleviated by reducing the absorbed doses. Uehara et al. (2007) incorporated a cleavable glycyl–lysine linker between ^188^Re and antibody fragments, which was cleaved at the brush border of proximal tubules. As a result, ^188^Re was not absorbed along with the antibody. The re-absorption of proteins can be lowered by co-injecting other molecules, including lysine, arginine, poly-l-lysine, succinylated gelatin, fractionated albumin, and albumin-derived peptide (Pimm and Gribben 1994). Another method is to inhibit the receptor-mediated endocytosis. For example, the renal uptake of ^111^In-octreotide was reduced by probenecid, which inhibits ochratoxin A transport and multidrug resistance proteins (Stahl et al. 2007).

## Nanoparticles for radiosensitization

Despite the improvement in imaging and radiation sources, it is still a great challenge to confine the curative dose of radiation within tumor tissue while sparing the adjacent normal tissues. In many cases, the radio-toxicities to non-tumor tissues remain the dose-limiting factors. Therefore, it is of great benefit to increase the sensitivity of tumor cells to radiation, so that lower radiation doses can be used to eradicate tumor. Up to date, a plethora of radiosensitizers have been developed and evaluated based on different mechanisms, such as enhancement of dose, generation of radical oxygen species (ROS), and alteration of biological responses to radiation. A brief discussion of nanoparticles equipped with radiosensitization capabilities are presented as follows.

### Dose enhancement using high atomic number (Z) materials

Dose enhancement during radiotherapy was pioneered by Matsudaira et al. (1980), where iodine (Z = 53) was found to increase the absorbed dose of X-rays in cell culture. Since then, the dose enhancement has been extended to various metal elements, including gold (Au, Z = 79) (Schuemann et al. 2016), gadolinium (Gd, Z = 64), platinum (Pt, Z = 78), and bismuth (Bi, Z = 83) (Chithrani et al. 2010; Kamiar et al. 2013; Jeremic et al. 2013; Alqathami et al. 2013; Yao et al. 2014; Ma et al. 2015; Le Duc et al. 2011; Miladi et al. 2013; Kryza et al. 2011; Miladi et al. 2015; Porcel et al. 2010; Usami et al. 2007, 2008). Notably, Gd-based nanoparticles presented both diagnostic and therapeutic potentials, since Gd also provides MRI contrast (Le Duc et al. 2011). Classical models have attributed the radiosensitization to the physical dose enhancement arising from the keV electrons. However, biological and chemical changes may also occur that subsequently increase the sensitivity of cells or tissues to radiation. The actual biological effects may come from both perspectives (Subiel et al. 2016).

Extensive studies have focused on exploring the underlying mechanisms of radiosensitization via high-Z metals (Porcel et al. 2010). An excellent overview was presented by Hainfeld et al. in their report of AuNP-enabled radiosensitization (Hainfeld et al. 2008). In general, high-Z atoms have large cross sections to absorb radiation energy, after which secondary radiations can be generated to cause damages in nearby DNA molecules. Such secondary radiations include short-range low-energy electrons, Auger electrons, photoelectrons, and characteristic X-rays (Jeremic et al. 2013). The ranges of such secondary electrons in tissue vary from about 10 nm for the Auger electrons, to 100 µm for the photoelectrons, and as far as several centimeters for the fluorescence photons (Hainfeld et al. 2008). Using gold as an example, the dose enhancement is restricted to the vicinity of gold atoms. It is critical to have a uniform distribution of gold throughout the whole tumor, or more preferentially close to DNA molecules. As a result, nanoparticle formulations are advantageous due to their ability to selectively deposit in tumor and to be internalized by tumor cells (Oh et al. 2011). Indeed, micrometric gold nanoparticles were restricted to the site of injection, instead of distributing throughout the tumor (Herold et al. 2000). It should be noted, however, majority of the nanoparticles reside within cytoplasm unless a specific nuclei-delivery is incorporated in the design of nanoparticles. It remains of question whether DNA damage can be induced by the secondary electrons produced from cytoplasmic nanoparticles. On the other hand, the dose enhancement factor (DEF) by AuNPs varies with radiation sources. A simulation study by Lin et al. (2015) calculated that 43% additional dose was produced by AuNPs for 250 keV photons, 1% for 6 MeV photons, and only 0.1% for protons. Rahman et al. (2014) irradiated 1.9-nm AuNPs with X-rays from 30 to 100 keV. The 40-keV X-ray had the highest DEF at 3.47, while both lower and higher energy X-rays had lower DEFs. A probable explanation is that low-energy photons can be absorbed by the K or L shell electrons of Au. The resultant secondary Auger electrons emission then causes further damage to DNAs. In contrast, high-energy photons tend to have Campton scattering without producing Auger electrons (Kobayashi et al. 2010).

Hainfeld et al. (2004, 2006, 2008) first examined the in vivo radiosensitization effect of 1.9-nm AuNP in a murine EMT-6 mammary tumor model. Mice were injected with a dose of 1.35 gAu/kg, which resulted in a 4.9 ± 0.6% ID/g tumor uptake at 5 min post injection. For radiation therapy, 30 Gy of 250 kV X-ray was applied to the tumor region at 2 min post injection of AuNP. Most tumors were undetectable at 1 month after treatment with AuNP plus radiation, while the monotherapy groups only delayed tumor growth. Long-term survival studies (up to 1 year) also showed a benefit of the combination therapy as well as a response dependent on the dose of gold (Hainfeld et al. 2004). While many studies have proven the radiosensitization effects of AuNPs in both cell culture and animal models, the long-term body retention of AuNPs remains a concern despite that AuNPs are often considered safe and bio-inert (Zelasko-Leon et al. 2015). To minimize such potential chronic toxicity, several clearable formulations of AuNPs were developed. Zhang et al. (2015) prepared 2.8-nm glutathione-protected AuNPs for radiosensitization. The combination therapy of AuNPs and 5 Gy of ^137^Cs radiation completely inhibited the growth of U14 brain tumor xenograft. Notably, the ultrasmall 2.8-nm AuNPs had minimal uptake in the RES organs such as liver and spleen, and were cleared via kidney. The body clearance of AuNPs was completed at 28 days after injection. The same group also studied the sub 2-nm glutathione-protected AuNPs (Zhang et al. 2014), showing similar radiosensitization effects. In this study, however, significant liver uptake (~30% ID/g) was found at 24 h post injection. Such discrepancy may arise from the fact that fluorescence dye Cy5 was conjugated to AuNPs and used as tracer for uptake studies. The dissociation and subsequent retention of Cy5 in liver may cause a falsely high uptake of AuNPs. Further studies are required to clarify such an issue.

Other metal elements were also tested for dose enhancement. Gadolinium-based (Gd, Z = 64) ultrasmall nanoparticles (GBNs) have been evaluated for radiosensitization by a group of French researchers (Le Duc et al. 2011; Miladi et al. 2013; Kryza et al. 2011; Miladi et al. 2015). The GBNs are composed of a Gd_2_O_3_ core surrounded by a silica shell, with a hydrodynamic diameter of 3.8 ± 0.1 nm. Biodistribution study found a high rate of renal clearance after the intravenous injection of GBNs. Over 95% of injected dose was eliminated within 18 days after injection, via both urine and feces. The small size also reduced the fenestration in organs of the mononuclear phagocytic system, showing a minimal uptake in liver (<0.5% ID/g) (Kryza et al. 2011). Le Duc et al. (2011) evaluated the GBNs in a rat model bearing intracerebral 9L gliosarcoma along with microbeam radiotherapy. DTPA was coated to the surface of GBNs to chelate Gd^3+^ ions and subsequently provide MRI contrast. MRI delineated the tumor up to 45 min after intravenous injection of GBNs, with a tumor-to-tissue ratio of 60.4 being reached at 20 min post injection. Microbeam radiotherapy was applied to tumor at 20 min post injection, and the mean survival time was extended to 90 days, compared to 47 days for radiation-only control, and 19 days for non-treatment control. Miladi et al. (2015) examined the GBNs in several radio-resistant human head and neck squamous cell carcinoma models along with 250 keV photon irradiation. The combination of GBNs and radiation overcame the radiation resistance in SQ20B stem-like cells, significantly delayed tumor growth with elevated late apoptosis and reduced cell proliferation. Sancey et al. (2014) reviewed in detail the evolution of Gd-based theranostic nanoparticles, which has recently been benchmarked as AGuIX^®^. It should be noted that high radiosensitization effects were reached even with a Gd concentration as low as 1 ppm in tumor. Auger electrons were believed to be the main contributor for radiosensitization under such scenarios.

The radiosensitization of platinum-based nanoparticles has been explored (Porcel et al. 2010; Usami et al. 2005, 2007, 2008, 2010), especially in combination with fast atomic ions such as Fe^26+^ and C^6+^. Unlike photons and electrons, such heavy atoms with large cross sections interact with tissues on their trajectory tracks, and therefore are more efficient than conventional radiations (Dan et al. 2003; Mozumder 2003). Porcel et al. (2010) showed that both Pt ions and 3-nm Pt nanoparticles bound to DNA molecules, and increased the number of single-strand breaks and double-strand breaks during radiation. The enhancement ratios were between 1.37 and 2.17. Importantly, DNA damages were significantly alleviated by adding radical scavengers, indicating that radiation-induced water radicals were a prominent culprit of DNA damage (Usami et al. 2007). Since DNA is generally accepted as the main target of ionizing radiation in cells, the produced DNA damages may serve as a probe to quantify the radiation-enhancing effect of nanoparticles. Gel electrophoresis has been used to quantify the number of single- and double-strand breaks ex vitro (Usami et al. 2007), while many staining techniques are available to visualize the DNA breaks in cell culture or tissues (Mah et al. 2010; Zhu et al. 2014).

Bismuth (Bi, Z = 83)-based nanoparticles have shown to induce above 80% enhancement of radiation dose while maintaining low toxicities (Alqathami et al. 2013). Yao et al. (2014) prepared Bi_2_S_3_-loaded PLGA nanoparticles. The resultant nanoparticles were 754.6 nm in size and distributed to PC3 prostate xenograft tumor within 30 min of injection, although 80% of injected dose was fenestrated in liver. The combination therapy with 6-Gy radiation and Bi_2_S_3_-PLGA inhibited tumor growth more effectively than monotherapy groups, along with the upregulation of apoptosis-related proteins, including p53, Bax, and Bcl-2. Ma et al. (2015) prepared Bi_2_S_3_-embedded mesoporous silica nanoparticles (BMSNs) for radiosensitization. Hydrophobic Bi_2_S_3_ nanoseeds of 2–3 nm in diameter were first prepared, followed by a sol–gel condensation of triethoxysilane, to form the mesoporous nanoparticles of 72.7 nm in size. BMSNs were used in combination with 1.85 MBq interstitial ^32^P irradiation to treat PC3 prostate xenograft tumor. Compared to monotherapies, the combination therapy effectively inhibited tumor growth and induced prominent apoptosis of tumor cells.

### Generation of reactive oxygen species (ROS)

Radiation-generated ROS is an important cytotoxic component for killing cancer cells (Allison et al. 2004). The cytotoxic ROS can also be generated via photodynamic therapy (PDT). PDT is an emerging non-invasive treatment that utilizes light-excitable photosensitizers to produce cytotoxic ROS upon photon illumination (O’Connor et al. 2009). Unlike many other chemotherapy drugs that need to enter cytoplasm or nucleus to function, the major target of ROS are lysosomes that belong in the endocytosis pathway of nanoparticles (Leamon and Low 1991). Therefore, endosome escape is not required for the ROS-generating nanoparticles to exert cell-killing effects. Once lysosomes are ruptured by ROS, cathepsin B or L are released to activate caspases, which in turn inactivates proteins that protect cells from apoptosis (de Castro et al. 2016). Conventional PDT has two drawbacks. First, the generation of ROS requires oxygen, and therefore has limited efficiency in the hypoxic tumor microenvironment (Vaupel et al. 2001). Second, traditional photosensitizers are excited by ultraviolet or visible lights that have limited tissue penetration (Allison et al. 2004).

Recently, semiconductor nanoparticles have been developed that can downconvert the X-ray energy into ultraviolet/visible region, which can subsequently activate the nearby photosensitizers (Bulin et al. 2013). Zhang et al. (2015) prepared 33-nm cerium(III)-doped LiYF_4_@SiO_2_@ZnO nanoparticles that can generate cytotoxic hydroxyl free radicals upon X-ray irradiation. The X-ray was downconverted to ultraviolet fluorescence in the cerium(III)-doped LiYF4 core, and further generated electron–hole (e^−^–h^+^) pairs in ZnO. Highly reactive hydroxyl radicals were produced from the reaction between the hole (h^+^) and surrounding water. Since no oxygen was involved in the reaction, free radicals were generated in both normoxic and hypoxic conditions. Importantly, the inorganic photosensitizer was resistant to photobleaching during X-ray treatment. The combination therapy of the nanoparticle with 8-Gy X-ray radiation effectively inhibited the growth of HeLa xenograft tumor up to 15 days after treatment.

Auger effects may increase ROS generation upon X-ray irradiation (Kobayashi et al. 2010). He et al. (2015) prepared mesoporous silica nanoparticles loaded with selenocysteine (SeC@MSNs). The ROS generated by SeC@MSNs and 2-Gy X-ray via Auger effects was 202% of control. Extensive cell apoptosis was recorded, evidenced by the sub-G1 population in cell cycle analysis. The activation of apoptosis pathways, e.g., p53 and ATM/ATR, was also observed. In vivo anti-tumor efficacy in HeLa xenograft showed that combination group had the smallest tumor volume. In a similar study, Huang et al. (2014) conjugated bovine serum albumin with phenylbenzo (Assmus 1995; Zoller et al. 2009; Li 2014) selenadiazole derivatives. The resultant nanoparticles also generated ROS upon X-ray irradiation, and radiosensitized HeLa xenograft tumor. Alternatively, nanoparticles can catalyze the production of hydroxyl radicals in aqueous solution upon X-ray irradiation (Sicard-Roselli et al. 2014).

It is known that the efficacy of radiotherapy can be mitigated by the hypoxic tumor microenvironment (Vaupel et al. 1991). Prasad et al. (2014) prepared MnO_2_-bound albumin nanoparticles (A–MnO_2_) to re-oxygenate the tumor microenvironment. The hypoxic tumor microenvironment and the highly proliferating tumor cells, together, produce ROS, e.g., H_2_O_2_. MnO_2_ reacts with the H_2_O_2_ to produce O_2_. Within 7 min of intratumoral injection of A–MnO_2_, the vascular saturated O_2_ increased by 45% compared to control. Immunohistochemical staining found decreased expression of both hypoxia markers HIF-1α and VEGF in treated tumor. When combined with radiation, a significant inhibition of tumor growth was observed, along with elevated staining of DNA double-strand breaks.

### Re-distribution of cell cycles to radiosensitive G2/M phases

While most radiosensitization experiments are conducted using keV-energy radiation sources, current clinical therapy has shifted toward higher energy (MeV) sources in order to treat deep-seated tumor, as well as to reduce toxicity to skin (Jeremic et al. 2013). However, the DEF of AuNPs is only marginal, between 1.1 and 1.2, for MeV X-rays. Nevertheless, a series of studies were performed on the combination of 6 MeV radiation and AuNPs of sizes between 13 and 55 nm (Liu et al. 2015; Li et al. 2015; Wang et al.^2015^). Although radiosensitization effects were still observed, the underlying mechanism was found not due to dose enhancement, but rather the re-distribution of cell cycles to the radiosensitive G2/M phases by AuNPs. Cyclin A, cyclin B1, cyclin E, and p53 are the critical mediators of AuNP-induced arrest of cell cycles (Roa et al. 2009). Liu et al. (2015) used EGFR-targeting hollow gold nanospheres to enhance the MeV radiation in cervical cancer. Uptake of hollow gold nanospheres induced cell arrest in G2/M phase (38.4 vs.10.2% in control), leading to increased cell apoptosis during radiation. In addition, the hollow gold nanospheres elevated the expression of pro-apoptotic regulator, which may also have contributed to the radiosensitization. Wang et al. (2015) combined thioglucose-modified AuNPs (16 or 49 nm) with 6 MeV X-ray irradiation for the treatment of MB-MDA-231 triple-negative breast cancer cells. Cell arrest in G2/M phases was observed in cells treated with AuNPs of both sizes. However, it is not clear how AuNPs caused cell arrest in G2/M phase, which remains to be studied. The 49-nm AuNPs had higher uptake in cancer cells, and subsequently had higher sensitive enhancement ratio (SER) at 1.86 than the 16-nm ones (SER = 1.49).

Chemotherapy drugs constitute another category of cell cycle regulators (Russo et al. 2016; Li et al. 2016), among which paclitaxel is widely used to treat various types of cancer (Pazdur et al. 1993). Paclitaxel promotes the assembly of microtubules and inhibits their disassembly (Pazdur et al. 1993). Cells treated with paclitaxel are predominantly arrested in G2/M phases, and therefore have increased sensitivity to radiation (Creane et al. 1999). Due to its limited water solubility, paclitaxel has been formulated into many nanoformulations to increase its bioavailability. Our group developed a poly(l-glutamic acid)-conjugated paclitaxel (PG-paclitaxel) (Milas et al. 2003; Ke et al. 2001; Li et al. 2000; Li et al. 2000). In the OCA-1 ovarian cancer xenograft model, PG-paclitaxel enhanced the radiation response by a factor of 7.2–8.4 (Milas et al. 2003). Recently, Werner et al. (Werner et al. 2013) compared the radiosensitization of free paclitaxel and PG-paclitaxel in non-small cell lung cancer models. PG-paclitaxel had higher sensitizer enhancement ratio (SER) in both A549 cells (1.23 vs. 1.12 for paclitaxel) and H460 cells (1.12 vs. 1.03 for paclitaxel). When combined with 5 daily fractions of 3-Gy radiation, PG-paclitaxel led to significantly longer delay of tumor growth than paclitaxel in the H460 xenograft model (*p* = 0.008). Similarly, Jung et al. (2012) prepared paclitaxel-loaded polymeric nanoparticles to radiosensitize A549 xenograft model. The resultant nanoparticles were 39.4 nm in size and actively internalized by cancer cells. The volume of tumor treated by nanoparticle and radiation was only 38.9% of un-treated tumor. Currently, PG-paclitaxel is under development by CTI Biopharma as paclitaxel poliglumex in combination with cetuximab and radiotherapy, to treat patients with head and neck cancer (https://clinicaltrials.gov). The study is ongoing and its results are pending.

### Disruption of DNA damage repair

Ionizing radiation creates DNA damages such as double-strand break, single-strand break, and altered bases (Gavande et al. 2016). The readers are referred to reference (Kavanagh et al. 2013) for a broader understanding of radiation-induced DNA damages. These DNA damages can be repaired via various pathways including non-homologous end joining, homology directed repair, base excision repair etc. Many protein regulators are involved, such as DNA-PK, DNA-ligases, Rad51, and ATM (Lord and Ashworth 2012). Many chemotherapy drugs can disrupt the DNA damage repair, and subsequently enhance radiotherapy (Wieringa et al. 2016). Several nanoparticle-based formulations for inhibition of DNA damage repair are discussed below.

Wang et al. (2015) prepared PLGA-based polymeric nanoparticles to encapsulate histone deacetylase inhibitors (HDACIs). HDACIs disrupt the repair of DNA double-strand breaks, and subsequently cause cell death (Chinnaiyan et al. 2005). However, the effect of HDACIs is reversible. A prolonged exposure to HDACIs is required to achieve successful tumor inhibition and radiosensitization (Lee et al. 2010). Wang et al. utilized the nanoformulations to achieve a controlled release of HDACIs in tumor, and therefore to extend the drug exposure. In vitro radiosensitization was confirmed via colony survival assays and immunostaining of γ-H2AX DSB foci. PC3 (prostate cancer) and SW620 (colon cancer) xenografts were treated with 3-Gy X-ray irradiation and the nanoparticles. Significantly slower tumor growth was observed in combination groups.

Au et al. (2015) prepared PEG–PLGA polymeric nanoparticles to encapsulate docetaxel and wortmannin. Docetaxel induces cell cycle arrest in G2/M phases, as well as increases ROS production (Rabi and Bishayee 2009). Wortmannin is a potent inhibitor for phosphoinositide 3-kinase (PI3-K), and disrupts the repair of DNA damages (Wipf and Halter 2005). The resultant nanoparticles were 36 ± 6 nm in size. Up to 40% of injected nanoparticles accumulated in tumor, while liver trapped around 60% of injected dose. When combined with 8-Gy X-ray radiation, the mean survival of H460 tumor-bearing mice was 33.2 days longer than non-treatment control, and 28 days longer than radiation-only group.

Recently, we have developed several cyclopamine-loaded nanoparticles for radiosensitization (You et al. 2015; Zhao et al. 2015). Cyclopamine is a potent inhibitor for sonic hedgehog signaling, and disrupts the repair of radiation-induced DNA damages. When testing pancreatic cancer cells, the cyclopamine-loaded polymeric micelles enhanced the radiation by a factor ranging from 1.5 to 1.8 along with the extended presence of γ-H2AX foci (Zhao et al. 2015). We also evaluated the radiosensitization of cyclopamine-loaded lipid nanoparticles in a pancreatic cancer xenograft (Miapaca-2) and a 4T1 breast cancer model. ^177^Lu-conjugated polymeric micelles were used as intratumoral radiation source. The combination therapy was more effective in tumor suppression than radiation monotherapy in both tumor models.

### Combination with photothermal therapy

It is well known that hollow gold nanostructures exhibited unique optical properties to absorb near-infrared light irradiation (Oldenburg et al. 1998), and transform its energy into heat (Liu et al. 2008). A myriad of nanoparticles with different chemical compositions and architectures have been developed during the past decades that exhibit photothermal conversion properties in the near-infrared region of light (Huang et al. 2008; Melancon et al. 2011; Song et al. 2015; Xiao et al. 2013).

The nanoparticle-mediated photothermal effects are confined to the close vicinity of nanoparticles, and therefore can minimize the damage to adjacent normal tissue. While the high-temperature thermal ablation results in acute cell necrosis, hyperthermia (40–46 °C) can induce cellular biochemical alterations that can synergize with radiation (Roti Roti 2008). Diagaradjane et al. (2008) reported that gold nanospheres induced hyperthermia in tumor upon laser irradiation. The tumor perfusion increased, resulting in fewer hypoxic regions. As a result, the tumor response to radiotherapy was significantly enhanced: the tumor doubling time was 29 days for the combination therapy group, compared to 17 days for the radiation monotherapy group. Atkinson et al. (2010) discovered that gold nanosphere-mediated hypothermia depleted the population of breast cancer stem cells. While radiation monotherapy enriched the cancer stem cells by 30%, the combination therapy with hyperthermia reduced their population by more than 70%. Histological analysis also revealed that the combination therapy-treated tumors had more differentiated phenotypes. CuS nanoparticles display strong absorption of near-infrared light, which enabled the photothermal ablation therapy. We recently examined [^64^Cu]–CuS nanoparticles for the dual radiotherapy (by β emission from ^64^Cu) and photothermal therapy against 4T1 breast cancer model (Zhou et al. 2015b). Photothermal therapy decreased the number of tumor mammospheres, indicating the tumor-initiating cells were depleted by photothermal therapy. In vivo animal study showed that combined photothermal therapy and radiotherapy mediated by [^64^Cu]–CuS induced better anti-tumor activity than either photothermal therapy (mediated by CuS nanoparticles plus near-infrared laser) or radiotherapy (mediated by [^64^Cu]–CuS nanoparticles) alone, as judged by significantly reduced number of lung metastasis nodules and prolonged survival of mice bearing orthotopic 4T1 breast tumor.

## Conclusions and future perspectives

We have reviewed the application of nanoparticles for internal radiotherapy and radiosensitization. One of the key premises of nanoparticles is to deposit as much radiation energy as possible to tumor and make tumor as vulnerable as possible to radiotherapy. Another basic feature of nanoparticles is their multi-functionality and the capability to enable multimodality therapy directed to the same treatment volume at the same time. It is expected that nanoparticle-based radiotherapy will make significant contributions to cancer treatment when successfully translated into the clinic. However, success in combining nanomedicine and radiotherapy in the clinic will require advances in multiple fronts. First, the tumor-specific accumulation of nanoparticles is still far from being ideal, with a majority of the injected dose in most nano-carriers being sequestered in organs of the mononuclear phagocytic system. Future studies are needed to direct more efforts toward developing nanoparticle systems with minimal retention in the body after radiotherapy is over. Second, reproducible large-scale production processes must be developed and implemented under good manufacturing practice (GMP) guidelines. In addition, the biosafety profiles of nanoparticles must be determined using the GMP products. These studies are often time-consuming and resource-intensive, and have become the bottleneck for clinical translation of any nanoparticle-based therapeutic agent. Radiolabeled nanoparticles for internal radiotherapy add another dimension of complexity, because radiolabeling often needs to be completed in short time while keeping a satisfactory labeling efficiency and radiostability. The dosimetry of radiolabeled nanoparticles also needs to be adequately determined to estimate potential radiation exposure to normal organs.
